# HIV tropism switch in archived DNA of HIV-HCV subjects successfully treated with direct-acting antivirals for HCV infection

**DOI:** 10.1038/s41598-021-88811-6

**Published:** 2021-04-29

**Authors:** Monica Basso, Daniela Zago, Renzo Scaggiante, Silvia Cavinato, Irene Pozzetto, Camilla Stagni, Beatrice Parisatto, Anna Maria Cattelan, Giuliana Battagin, Loredana Sarmati, Saverio Giuseppe Parisi

**Affiliations:** 1 Department of Molecular Medicine, University of Padova, Via Gabelli 63, 35100 Padua, Italy; 2 Infectious Diseases Unit, Belluno Hospital, Belluno, Italy; 3 Infectious Diseases Unit, Azienda Ospedaliera-Universitaria di Padova, Padua, Italy; 4 Clinical Infectious Diseases, Vicenza Hospital, Vicenza, Italy; 5 Infectious Diseases Clinic, Università Tor Vergata, Rome, Italy

**Keywords:** Diseases, Medical research, Pathogenesis

## Abstract

We described short-term HIV tropism changes occurring in peripheral blood mononuclear cells and the correlations with HIV DNA value in HIV-HCV co-infected patients cured for HCV disease and with undetectable HIV viremia or residual viremia (RV). Plasma HIV RNA, cellular HIV DNA and tropism were evaluated pre-HCV treatment (baseline, BL) and at 12(T1) and 24(T2) weeks after HCV treatment start. V3 sequences were interpreted using Geno2pheno and classified as R5 only if all three sequences had an FPR ≥ 10% and as X4 when at least one replicate sequence had an FPR < 10%. Forty-nine patients (21 with X4 and 28 with R5 virus) were enrolled. Five X4 patients and 9 R5 subjects experienced at least one tropism change,11 with RV:1/5 patients with X4 infection at BL switched at T1 versus 8/9 in the R5 group (p = 0.022977) and the difference was confirmed in subjects with RV (p = 0.02);6/9 R5 patients switching at T1 confirmed the tropism change at T2. No significant differences in HIV DNA values between patients with RV starting with a R5 or X4 tropism and experienced tropism switch or not were found. Short-term tropism switch involved almost a third of patients, in all but three cases with HIV RV. Being R5 at BL is associated to a higher instability, expressed as number of tropism changes and confirmed switch at T2.

## Introduction

As the human immunodeficiency virus (HIV) and hepatitis C virus (HCV) have common routes of transmission, their co-infection is estimated to affect 5–7 million people worldwide^1^.

The advent of therapy with direct-acting antivirals (DAA) has greatly modified HCV therapy efficacy in HIV-HCV co-infected subjects, who have now achieved sustained virological response (SVR) rates exceeding 95% and comparable to those of HCV mono-infected subjects^2^, as there was liver function improvement after HCV clearing^3^.

However, HCV clearance obtained with DAA treatment may cause an imbalance in the immune system. We previously described a higher percentage increase in cellular HIV DNA in co-infected subjects with HCV clearance and undetectable plasma HIV viremia with respect to those with HIV low-level viremia in a 12-week study period^4^ as well as a decrease of soluble CD163 and of soluble CD14 in plasma independent from HIV RNA detection and of cellular HIV DNA value in a 24-week period^5^, suggesting a complex figure of HIV archive in relation to HCV viremia perturbation.

The chemokine receptors CXCR4 (X4) and CCR5 (R5) are used to enter target cells; they play an important role in HIV pathogenesis and response to anti-viral treatment and disease progression^6–8^. Furthermore, non R-5 tropism correlates with higher HIV DNA values^9^ and to non-AIDS events development^10^. HIV tropism may also evolve at very low level of HIV viremia and during the successful plasma HIV RNA suppression^11,12^—now a common virological figure during anti-retroviral therapy (ART). HCV infection does not seem to influence co-receptor tropism, nor is specific co-receptor use associated with HCV infection^13^. Plasma HCV RNA values are comparable between patients infected with R5 and X4 viruses^14,15^.

The present study aimed to describe short-term HIV tropism changes in the peripheral archive and correlations with HIV DNA value in HIV-HCV co-infected patients cured for HCV disease according to HIV disease control (undetectable HIV viremia versus residual viremia).

## Methods

Patients with HIV-HCV co-infection successfully treated with DAA, aged more than 18 years and with sub-type B HIV-1 infection were included in the study if they fulfilled the following criteria: (1) hepatitis B surface antigen negativity; (2) no previous treatment with CCR5 antagonists; (3) CD4+ cell count ≥ 200 cells/mm^3^ and successful ART ongoing when anti-HCV therapy started; (4) HCV RNA undetectable at week 12 (or < 12 IU/ml at week 12, but undetectable at week 16 and undetectable at W24 of study time regardless of anti-HCV treatment programmed length; (5) no plasma HIV RNA value ≥ 100 IU/ml^16^ in all tests performed during the study period. Ongoing ART regimen and HIV drug combination modifications due to drug-drug interactions were chosen by the treating physician according to the current guidelines.

Three main study points were identified: baseline (BL) corresponding to pre-HCV treatment, T1 (week 12 of anti-HCV treatment) and T2 (week 24 after HCV treatment started); T2 corresponded to the end of treatment in case of a 24-week schedule and to SVR at week 12 of follow-up in case of a 12-week schedule. Plasma HIV RNA, cellular HIV DNA and tropism were all evaluated at BL, T1 and T2; at a fourth time point (T3, week 48 after HCV treatment started) plasma HIV RNA was available for all the subjects and tropism for 26 patients.

Patients were classified as having suppressed plasma HIV viremia, if plasma HIV RNA was undetectable (undetectable viremia, UV) and as having residual viremia (RV) when no plasma HIV RNA value was ≥ 100 copies/ml during the study period and in the year before the anti-HCV treatment started. Patients with plasma HIV RNA value ≥ 100 copies/ml were excluded.

HCV genotype was determined with VERSANT HCV genotype 2.0 assay (INNOLiPA, Innogenetics, Ghent, Belgium); in-house sequencing of the core region (nt 429–741) was used as the confirming method. HCV RNA was tested with Abbott Real-Time HCV assay (Abbott Molecular, Des Plaines, IL, USA) and had a lower limit of quantification of 12 IU/ml.

The Abbott Real-Time HIV assay (Abbott Molecular, Des Plaines, IL, USA) was used for plasma HIV RNA value determination; the lower limit of quantification was 40 copies/ml.

The liver fibrosis degree was evaluated with transient elastography (FibroScan, Echosens, Paris, France) or by liver biopsy, according to the Metavir score^17^.

The patients gave informed written consent to inclusion in the study and to the use of their anonymized data for scientific aims. This study was conducted in accordance with the Helsinki Declaration and local legislation and it was approved by the Ethics Committee of Padova University Hospital (prot. 2606-12P).

### Genotypic prediction of viral tropism

The genotypic analysis of HIV tropism was conducted as previously described on peripheral blood mononuclear cells (PBMC)^11,18^. In summary, nested PCR with 1F1 and 1R1 as the outer primers and 3F3 and 2R2 as the inner primers were used to amplify the V3 sequences. The number of ambiguities examined before running Geno2pheno was controlled and if the number was greater than two they were defined as relevant. In that case, we repeated the test and excluded the sample if the ambiguities were always present. Two samples met this criteria and the two patients were excluded from the study. Sequencing was repeated when a single or double ambiguity gave discordant results in terms of false positive rate (FPR). The generated V3 sequences were then interpreted using the bioinformatic tool Geno2pheno available at http://coreceptor.bioinf.mpi-inf.mpg.de (accessed by October 2020)^19^.

Longitudinal discordant results were tested twice (i.e. three amplifications were performed twice) to confirm the tropism switch. Similarly, all samples with FPR ranging from 5 to 20% were confirmed by a second analysis starting from sample amplification. Briefly, we defined a clear viral switch only with at least two concordant predictions based on a triple sequence tool. Samples were classified as R5 only if all three sequences had FPR ≥ 10%, while samples were classified as X4 when at least one replicate sequence had FPR < 10%^20,21^. The decision to apply the threshold of 10% was made to certainly identify the subjects with X4 infection and/or a clear change of the figure. In addiction to this classical approach, we performed a secondary analysis restricted to tropism evolution by identifying tropism with the mean FPR value (mFv) of the three sequences performed for each tropism determination:subjects with a (mFv) < 10% were classified as having a X4 virus infection. and as having a R5 virus infection otherwise (mFv) rule.

### Cellular HIV DNA quantitation

The cellular HIV DNA copy number in PBMCs was quantified according to the real-time TaqMan protocol^22^. A standard curve with a sensitivity of 5 copies/million PBMCs^23^ was built using the cell line 8E5 (containing 1 copy of integrated HIV DNA in each cell).

We always evaluated together in the same experiment BL, T1 and T2 samples of a specific patient and in duplicate both for HIV DNA and beta globin. When the difference between the two results was > 10% the test was repeated and the mean of the two results was the data included in the study. We conducted a duplicate analysis for both HIV and beta globin evaluation within the same experiment and calculated the mean of these values.

### Statistical analysis

The continuous variables recorded were age (years), CD4 positive cell count and percentage at BL, T1 and T2, HCV RNA (IU/ml), cellular HIV DNA (copies/10^6^ PBMCs); they were expressed as median value and inter-quartile range. Qualitative variables were gender, tropism in PBMC, fibrosis stage, reported as absolute value and percentage.

Chi-squared, Fisher’s exact, Mann–Whitney and Friedman tests were used as appropriate.

All p values were two-tailed and statistical significance was defined as p < 0.05.

Statistical analysis was performed using MedCalc Statistical Software version 19.6 (MedCalc Software Ltd, Ostend, Belgium; https://www.medcalc.org; 2020).

## Results

Fifty-five patients had BL tropism, but six were excluded from the study. Amplification and sequencing of the V3 region failed in one patient, two subjects had tropism test with ambiguities, while three did not perform the visit corresponding to T2. Most of the 49 patients included in the study were males (79.6%), the median age was 52 years (IQR 51–55 years) with a median CD4 cell count at BL as 550 cells/mm^3^ (IQR 320–642 cells/mm^3^) and a diagnosis of advanced liver fibrosis (85.7%). The most frequently prescribed anti-HCV regimen was ombitasvir + paritaprevir + dasabuvir + ribavirin and ledipasvir + sofosbuvir + ribavirin (seven patients for each, 30.4%) in the 12-week schedule and daclatasvir + sofosbuvir + ribavirin (nine patients, 34.6%) in the 24-week schedule (Table 1).

**Table 1:** Description of HCV drug regimens prescribed for 12 weeks (23 patients) or for 24 weeks (26 patients).

Anti-HCV treatment	12-week regimen (23 patients)	24-week regimen (26 patients)
OMV + PAR + DBV	1 (4.3)	0
OMV + PAR + DBV + RBV	7 (30.4)	5 (19.2)
DAC + SOF	1 (4.3)	0
LDV + SOF	6 (26.1)	6 (23.1)
LDV + SOF + RBV	7 (30.4)	3 (11.5)
SOF + RBV	1 (4.3)	0
OMV + PAR + RBV	0	3 (11.5)
DAC + SOF + RBV	0	9 (34.6)

Data are expressed as absolute number and percentage (respect to the number of subjects included in the regimen).

*OMV* ritonavir boosted ombitasvir, *PAR* paritaprevir, *DBV* dasabuvir, *RBV* ribavirin, *DAC* daclatasvir, *LDV* ledipasvir, *SOF* sofosbuvir.

At BL, 21 patients had X4 tropic virus infection and 28 had R5 tropic infection; the main viro-immunological characteristics of the two groups of subjects are comparable (Table 2). About half of the subjects had (25, 51%) modified ART before the anti-HCV therapy; two nucleoside reverse transcriptase inhibitors associated to one non-nucleoside reverse transcriptase inhibitor was the most frequent ongoing drug regimen at the start of anti-HCV therapy (13 patients, 26.5%). A detailed description of ART ongoing when anti HCV started is reported in Table 3. The number of patients who experienced tropism switch was comparable in subjects who modified ART (five out of 25, 20%) and in those who had ART unchanged (nine out of 24, 37.5%, p = 0.1797) and in those treated with a DDA regimen including ribavirin or not (9 out 35, 25.7% vs 5 out 14, 35.7%, p = 0.5).

**Table 2:** Characteristics of the HIV HCV patients with X4 tropic virus and with R5 tropic virus infection at baseline (BL).

	Patients with X4 infection (21, 42.9%)	Patients with R5 infection (28, 57.1%)	*p* value
Age (years)^a^	53 (50–56)	52 (51–55)	0.8789
CD4+ cells/count (cells/mm^3^) at BL^a^	551 (305–610)	540 (320–655)	0.6935
Percentage of CD4+ cells at BL^a^	27 (20.5–37.5)	30.5 (23–35)	0.9677
CD4+ cells/count (cells/mm^3^) at T1^a^	511 (255–702)	462 (314–716)	0.8875
Percentage of CD4+ cells at T1^a^	30 (19–40.2)	31.5 (23–37.5)	0.8398
CD4+ cells/count (cells/mm^3^) at T2^a^	546 (355–664)^b^	468 (312–655)^c^	0.6369
Percentage of CD4+ cells at T2^a^	30.5 (23–35)^b^	30 (25.2–36.5)	0.7763
HCV-RNA at BL (IU/ml)^a^	722,986 (77,626–1,911,151)	1,399,163 (150,922–4,198,566)	0.4921
HCV genotype 1, n (%)	12 (57.1)	14 (50)	0.6236
HCV genotype 2, n (%)	1 (4.8)	0	0.4285
HCV genotype 3, n (%)	2 (9.6)	8 (28.6)	0.1554
HCV genotype 4, n (%)	6 (28.6)	6 (21.4)	0.5690
DAA regimen including ribavirin, n (%)^b^	14 (40)	21 (60)	0.5271
DAA regimen not including ribavirin, n (%)^c^	7 (50)	7 (50)	
Residual viremia, n (%)	13 (61.9)	22 (78.6)	0.2059
Modified ART, n (%)	12 (57.1)	13 (46.4)	0.4624
HIV DNA in patients with residual viremia, (copies/PBMC)^a^	127 (90–245)	138 (68–261)	0.6572
HIV DNA in patients with suppressed viremia (copies/PBMC)^a^	36 (3–47)	116 (42–154)	0.1538

^a^Median and interquartile range.

^b^Percentage calculated with respect to the 35 patients treated with a DAA regimen including ribavirin.

^c^Percentage calculated with respect to the 14 patients treated with a DAA regimen not including ribavirin.

*DAA* direct-acting antivirals, *PBMC* peripheral blood mononuclear cells, *ART* antiretroviral therapy, *T1* week 12 of anti-HCV treatment, *T2* week 24 after HCV treatment started.

**Table 3:** Antiretroviral drug regimens ongoing before anti HCV treatment start.

Antiretroviral therapy regimen	Patients, n (%)
2 NRTI + 1 NNRTI	13 (26.5%)
2 NRTI + InSTI	8 (16.3%)
1 NRTI + InSTI	7 (14.3%)
2 NRTI + 1 PI (/PIr)	7 (14.3%)
PI (/PIr) + InSTI	6 (12.2%)
Other^a^	4 (8.2%)
1 NRTI + 1 PI (/PIr)	2 (4.1%)
1 PI	2 (4.1%)

For each drug combination are reported the absolute number of patients treated and the percentage respect to the total number of patients.

*NNRTI* non-nucleoside reverse transcriptase inhibitor, *PI* protease inhibitor, *Pir* ritonavir boosted protease inhibitor, *InSTI* integrase strand-transfer inhibitor, *NRTI* nucleoside reverse transcriptase inhibitor, *MRV* maraviroc.

^a^Other: MRV + INSTI + NRTI (1 pt); 1 PI + 1NNRTI (1 pt); 2 NRTI + MRV (1 pt); 1 NNRTI + INSTI (1 pt).

Both X4 and R5 patients with RV had higher HIV DNA values than those with UV but the statistical significance was achieved only in the X4 subjects (p = 0.0204).

Median values of CD4+ cells decreased significantly from BL to T2 in patients with RV starting with X4 tropic virus infection and experienced a tropism switch (590 cells/mm^3^, IQR 567–830 cells/mm^3^ at BL, 570 cells/mm^3^, IQR 570–667 cells/mm^3^ at T1 and 546 cells/mm^3^, IQR 497–560 cells/mm^3^ at T2, p = 0.04), even if the cell count was always higher than 500 cells/mm^3^.

### Tropism evolution (according to FPR < 10%)

Overall, 28.6% of subjects experienced at least one tropism change with respect to BL during the study period; five (23.8%) out of the 21 X4 patients and nine (32.1%) of the 28 R5 subjects. Nine of the 14 (64.3%) switching subjects experienced the only or the first switch at T1. Switching subjects with UV were one in the X4 group and two in the R5 group. The pattern of tropism change was different in patients with X4 or R5 virus infection at BL; six out of nine R5 patients reported a switch at T1, which was confirmed at T2. No X4 subject had this figure (p = 0.030969), but only isolated switches to R5 (one at T1 and four at T2). Moreover, the switch burden (defined as any switch identified, both isolated and confirmed) was 83.3% in R5 subjects (15 tests different from the tropism identified at BL: 8 at T1, 6 confirmed at T1, 1 late, at T2) and 50% in X4 patients (five tests different from the tropism identified at BL).

Furthermore, the timing was different: only 1/5 patients with X4 tropic infection at BL switched at T1 versus 8/9 in the group with R5 virus (p = 0.022977). The significant difference is also confirmed in the analysis restricted to subjects with detectable viremia (1/4 in X4 group versus all 7 R5 patients, p** = **0.02). A detailed description of tropism changes is reported in Table 4.

**Table 4:** Description of tropism changes at T1 and T2: patients are classified according to X4 tropic or R5 tropic virus infection at baseline.

	HIV RNA control	Tropism at BL	Tropism at T1	Tropism at T2
**Patients with X4 infection at baseline**				
Patient 1	UV	X4	X4	**R5**
Patient 2	RV	X4	X4	**R5**
Patient 3	RV	X4	**R5**	X4
Patient 4	RV	X4	X4	**R5**
Patient 5	RV	X4	X4	**R5**
**Patients with R5 infection at baseline**				
Patient 1	UV	R5	**X4**	**X4**
Patient 2	UV	R5	R5	**X4**
Patient 3	RV	R5	**X4**	R5
Patient 4	RV	R5	**X4**	**X4**
Patient 5	RV	R5	**X4**	R5
Patient 6	RV	R5	**X4**	**X4**
Patient 7	RV	R5	**X4**	**X4**
Patient 8	RV	R5	**X4**	**X4**
Patient 9	RV	R5	**X4**	**X4**

Bold characters correspond to tropism modification respect to BL.

*RV* residual HIV viremia, *UV* undetectable HIV viremia, *BL* baseline, *T1* week 12 of anti HCV treatment, *T2* week 24 after HCV treatment start.

**Figure 1:**
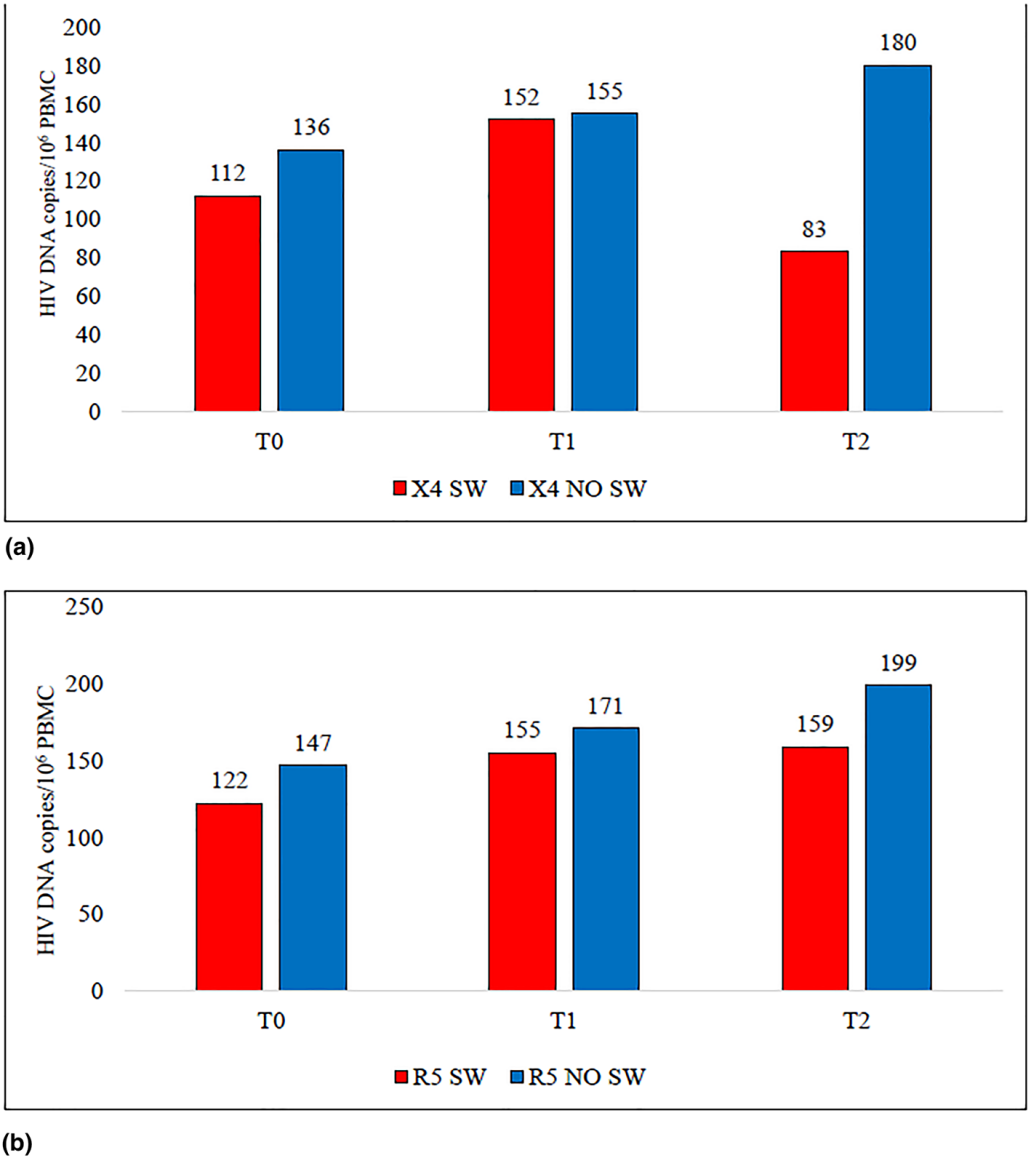
Description of median HIV DNA values (copies/10^6^ PBMC) at baseline, T1 and T2 in patients with residual HIV viremia bearing a X4 virus at baseline (**a**) or R5 virus at baseline (**b**) according to the detection or not of tropism switch. *PBMC* peripheral blood mononuclear cells, *T1* week 12 of anti-HCV treatment, *T2* week 24 after HCV treatment started.

No significant differences in HIV DNA values between patients with RV starting with a R5 or X4 tropism and experienced tropism switch or not were found, possibly due to the low number of subjects included in each group. However, patients with RV showed an increase in HIV DNA value from BL to T1 and from T1 to T2 in the case of stable tropism and an increase from BL to T1 and a decrease from T1 to T2, both in subjects starting with X4 infection and in those starting with R5 infection (Fig. 1).

A longer follow-up of tropism evaluation, at week 48, was available for 26 patients; 24 subjects (92.3%) had the same co-receptor use found at BL (4 of them after a switch), specifically, all the 10 subjects who had a X4 tropic virus and 14/16 of the patients with a R5 virus infection. Only two patients who experienced the switch at T1 and confirmed it at T2 confirmed the change at T3, differently from the four patients (2 X4 and 2 R5 at BL) who showed a single switch. Tropism detected at T2 did not predict HIV viremia levels at T3: both suppressed and viremic subjects at T2 changed their virological status in a comparable figure.

### Tropism in study population according to mean FPR value (mFv) rule

Overall, 23 tropism identification out of 147 (15.6%) changed when the (mFv) was applied: 15 patients (30.6% out of 49 pts) modified tropism classification in one study point at least and they included a higher number of subjects who experienced a switch with respect to the 34 subjects with immodified tropism when mFV rule was applied (6 subjects versus 10 subjects in the former, p = 0.0019).

In the 15 patients group an isolate tropism change occurred in 7 patients and a double modification in 8 subjects: in detail, when mFv rule was applied, 7 patients conventionally classified as with X4 stable tropism experienced a switch, 5 patients previously classified as switching turned to a stable R5 tropism and 3 subjects confirmed the figure of switchers with a different pattern.

A compared description of tropism results obtained with the conventional and mFv rule in the 15 patients is reported in Table 5.

**Table 5:** Description of tropism results obtained with the conventional rule (FPR < 10% corresponding to X4 tropic virus) and with the mean FPR value (mFv) rule (mean FPR value of the three sequences performed for each tropism determination < 10% corresponding to X4 tropic virus).

Conventional rule BL	Conventional rule T1	Conventional rule T2	mFv rule BL	mFv rule T1	mFv rule T2	Tropism changes with mFV rule	Number of changes with mFV rule	Tropism switches with conventional rule	Tropism switches with mFV rule
R5	X4	R5	R5	R5	R5	From X4 to R5 at T1	1	Yes	NO
X4	X4	X4	X4	X4	R5	From X4 to R5 at T2	1	No	YES
R5	X4	X4	R5	R5	R5	From X4 to R5 at T1 and T2	2	Yes	NO
X4	X4	X4	R5	X4	R5	From X4 to R5 at BL and T2	2	No	YES
X4	X4	X4	R5	R5	X4	From X4 to R5 at BL and T1	2	No	YES
R5	X4	X4	R5	R5	R5	From X4 to R5 at T1 and T2	2	Yes	NO
X4	X4	X4	R5	X4	X4	From X4 to R5 at BL	1	No	YES
X4	X4	R5	R5	X4	R5	From X4 to R5 at BL	1	Yes	yes
X4	X4	R5	R5	X4	R5	From X4 to R5 at BL	1	Yes	yes
X4	X4	X4	X4	R5	R5	From X4 to R5 at T1 and at T2	2	No	YES
X4	X4	X4	R5	R5	X4	From X4 to R5 at BL and at T1	2	No	YES
X4	X4	R5	R5	R5	R5	From X4 to R5 at BL and at T1	2	Yes	NO
R5	X4	X4	R5	R5	R5	From X4 to R5 at T1 and at T2	2	Yes	NO
R5	X4	X4	R5	R5	X4	From X4 to R5 at T1	1	Yes	yes
X4	X4	X4	R5	X4	X4	From X4 to R5 at BL	1	No	YES

Capital letters were used to describe a different tropism evolution throughout study time when mFv rule is applied.

## Discussion

In this study, we described the peculiar characteristics of short-term tropism switch occurring in PBMC of HIV-HCV co-infected patients, cured for their HCV disease with DAA focusing on the timing of the phenomenon and on the differences observed in patients with X4 or R5 virus infection before the HCV treatment started.

Tropism switch involved almost a third of patients, in all but three cases with HIV RV: this phenomena may occur as a stochastic events during HIV replication^24^ but we believed that HCV RNA clearance and not random mistakes of reverse transcriptase had a main role. A comparable rate of tropism switch in patients with undetectable viral load (defined as < 50 copies/ml or below the detection limit) and in those with persistently detectable viremia (median value of detectable viremia 9900 copies/ml) (19% and 18% respectively) was described by Castagna et al.^12^ in a cohort of 195 subjects on ART tested with a median interval of 22.9 months. Furthermore, short periods of HIV replication not enough to drive the HIV tropism evolution, as described by Baroncelli et al.^25^ in a study of 2 years of structured treatment interruptions (of five interruptions of 1, 1, 2, 2, and 3 months, separated by four periods of 3-month therapy). No data on HCV infection was included in this last work while in the Castagna’s study^12^ HCV infection had no unadjusted and adjusted relative risk in switch, but only 34.9% of the subjects enrolled were HCV positive and the infection was identified only by serology with no data on HCV RNA. Conversely, patients included in our study were all HCV RNA positive at BL, with undetectable or below the limit of quantification HCV viremia at T1 and all with negative HCV RNA at T2.

Most switches were identified at the time of the first detection of HCV RNA clearance: on this basis, we can hypothesize that ongoing HCV-related immune activation could have a major role in favoring the tropism switch process in patients with low-level HIV viremia.

The modifications in HCV RNA plasma level were associated with a significantly higher number of switches occurring at T1 and confirmed at T2 in patients with R5 virus infection at BL with respect to patients bearing an X4 virus, suggesting that the X4-archived virus population could be more involved in short-term viro-immunological modifications correlated to HCV clearance induced by DAA and its reappearance overwhelmed the R5 population. We have no definite explanation for this result. We hypothesize that reshaping of naive and central memory CD4+ T cells harboring X4 species could be involved; CXCR4-using HIV strains predominate in these cells and there is a negative correlation between HCV RNA level and CD4 central memory frequency in HIV-HCV patients, who had a lower number of naive CD4+ cells with respect to HIV mono-infected subjects^26–28^. However, we cannot exclude the involvement of mechanism other than HCV cure, as heterozygosis for the CCR5D32^29,30^.

Interestingly, we observed a high frequency of switches in the group of 15 patients who modified one virus tropism at least during the study period when mean FPR rule was applied (8 with the standard classification and 10 with the new approach). In our opinion the inclusion of the description of tropism picture identified by the mean could better describe the biological characteristics of HIV infection in a pilot study as ours, which aimed to describe a phenomena and not to select patients for CCR5-antagonist treatment. We observed a greater proportion of R5 using virus detected with this unconventional approach and this result is in accord with our opinion that the clinical implications of the change in co-receptor specificity into X4 found with the conventional method is not definitely an unfavorable event, even if any analysis of clinical implication is beyond the aim of this study.

First, the negative effects related to the emergence of X4 variants (rapid decline in CD4+ cell count, lower rates of survival, fast disease progression, non-AIDS events development) were reported after many months^10,31–33^ or in patients with primary infection^34^ and they involve primarily the HIV plasma viremia, that is to say people with an active replication. Second, HIV-HCV subjects who obtained SVR had a significant reduction in liver and non-liver-related complications and survival increase even in case of previous diagnosis of severe liver fibrosis^35–37^.

However, HCV-related residual inflammatory activity can occur in some subjects with HCV clearance: immune dysfunctions involving gamma delta T cells^38^ and CD8 + T cells^39^ persisted. HIV disease burden was not limited to plasma HIV viremia, but included HIV DNA, corresponding to the viral reservoir in long-lived cells^40–42^ that is constantly replenished by a low-grade viral replication in the lymphoid tissue^43^. For this reason, we included cellular HIV DNA quantification in the study.

We found no difference in cellular HIV DNA between patients with X4 or R5 tropic HIV infection at BL, T1 and T2; this lack of correlation differs from the data reported by Lombardi et al.^9^, who described that subjects with non-R5 virus had a higher HIV DNA reservoir in a cross-sectional work. The authors^9^ included about 40% of subjects classified as having HCV co-infection, but no further description was included, while we performed HIV DNA testing in patients who all had active HCV infection (at BL), with absent or minimal HCV RNA detection (at T1) and who all were HCV RNA negative from 2 months at least (T2). The different approach to plasma HIV RNA categorization could also explain the different results obtained in Lombardi’s work^9^ and in our study: the former had plasma HIV RNA < 50 copies/ml for > 6 months as inclusion criteria, while we categorized patients on the basis of HIV disease virological control in the year before the anti-HCV therapy started and during the 24 weeks of study time.

Our study has four main limits. The first was the short follow-up after DAA action on HCV, with a fourth study time at 48 weeks available only for a group of patients. The second was the lack of a control group both of HIV-HCV patients untreated for HCV infection and of HIV monoinfected patients. The third was the treatment with DAA regimen no more included in updated guidelines^44^: nevertheless, the subjects achieved SVR, which was the expected result. However, we confirmed tropism changes at T3 in R5 subjects who switched to X4 at T1 and conformed the result at T2; we are aware that a longer interval between HCV RNA clearance and tropism testing will be necessary to assess if the switch will have clinical relevance. However, this is a preliminary study and we could not predict the results obtained because data on repeated tropism testing are scarce and with different designs^45^. The forth was the lack of a group of HIV-HCV or at least HIV mono infected patients with tropism tested at the same study intervals. The study population included 49 subjects, all treated with DAA: no control group was tested because it was unethical to postpone anti-HCV therapy for 6 months and it was difficult to recruit many HIV monoinfected subjects to collect so frequently only for research aims and because there is evidence of long-term efficacy of ART regimen including maraviroc, an antagonist for CCR5, with extremely rare detection of switch to X4 tropism^46^. The small cohort size was composed of two groups of comparable numerosity and immuno-virological characteristics at BL and this is a strength of the study. The percentage of subjects with a X4 tropic virus infection at BL is high (42.9%) but comparable to that reported in previous studies in patients on successful ART^13,47,48^ and possibly influenced by the immune activation state^49^.

This longitudinal study had an original design with respect to previous works because it included multiple tropism tests modeled on HCV RNA decay under DAA treatment; previous studies on tropism evolution had longer tropism testing intervals^11,50–52^ and did not include subjects on anti-HCV therapy: however, the lack of control groups of HIV HCV coinfected patients and of HIV monoinfected subjects made our conclusions very hypothetical We observed HIV tropism switch in about a third of HIV-HCV subjects under successful anti-HCV therapy, that suggests this phenomenon also occurred in favorable clinical settings. Further studies are needed to confirm this result on a larger cohort of patients and to better characterize the sub-group of patients with modified tropism, applying next-generation sequencing approach to PBMC and RV to evaluate whether X4 tropic virus replication is ongoing.

## Data Availability

All data generated or analysed during this study are included in this published article.
